# CT-guided sacroiliac joint injection for chronic sacroiliitis: outcomes and predictors in a retrospective cohort study

**DOI:** 10.1007/s11547-026-02191-3

**Published:** 2026-03-11

**Authors:** Mostafa Farouk Balbaa, Antonio Barile, Mohamed Mahmoud El Shafei, Hoda Mohammed Aly Abdel Naby, Anna Maria Ierardi, Mohamed Ragab Nouh, Tarek El Sakka, Pierpaolo Biondetti, Francesco Arrigoni, Gianpaolo Carrafiello, Mamdouh Ahmed Zidan

**Affiliations:** 1 Department of Radiodiagnosis and Intervention, Kafrelsheikh University, Kafrelsheikh, Egypt; 2 Department of Biotechnological and Applied Clinical Sciences, University of L’Aquila, L’Aquila, Italy; 3 Department of Radiodiagnosis and Intervention, Alexandria University, Alexandria, Egypt; 4 Deapartment of Physical Medicine, Rheumatology and Rehabilitation, Alexandria University, Alexandria, Egypt; 5 Operative Unit of Radiology, Fondazione IRCCS Ca’ Granda Ospedale Maggiore Policlinico Di Milano, Milan, Italy; 6 Department of Emergency and Interventional Radiology, San Salvatore Hospital, L’Aquila, Italy; 7 Department of Health Sciences, Università Degli Studi Di Milano, Milan, Italy

**Keywords:** Sacroiliac joint, CT-guided injection, Low back pain, Sacroiliitis, Pain management, Functional outcome

## Abstract

**Purpose:**

To evaluate the safety, technical success, and clinical outcomes of CT-guided sacroiliac joint (SIJ) injections in patients with chronic sacroiliitis and to assess the impact of disease etiology, age, and body mass index (BMI) on therapeutic response.

**Materials and methods:**

This retrospective study included 80 patients with clinically and radiologically confirmed sacroiliitis who underwent CT-guided intra-articular SIJ injection between February 2020 and May 2022. Pain and disability were assessed using the Numerical Rating Scale (NRS) and the Oswestry Disability Index (ODI) at baseline and at 1-week, 1-month, and 6-month follow-up intervals. Patients were stratified by etiology (inflammatory vs degenerative), age, and BMI.

**Results:**

All procedures were technically successful and complication-free. Mean NRS scores improved from 6.86 ± 1.20 at baseline to 2.26 ± 0.78 at 6 months (*p* < 0.001). ODI scores decreased from 37.28 ± 9.84 to 5.68 ± 2.49 (*p* < 0.001). Greater ODI improvement was observed in inflammatory cases compared to degenerative ones (*p* < 0.05), though both subgroups showed significant clinical benefit. Age and BMI did not significantly affect outcomes.

**Conclusion:**

CT-guided SIJ injection is a safe and effective intervention for sacroiliitis, yielding sustained pain relief and functional improvement across both inflammatory and degenerative etiologies, independent of age and BMI.

## Introduction

Sacroiliac joint (SIJ) dysfunction is a frequently overlooked cause of chronic low back pain (LBP), with reported prevalence reaching up to 30% in the general population and among post-lumbosacral fusion patients [1, 2]. Its underdiagnosis stems from overlapping symptoms with lumbar spine pathology and the complex anatomy of the SIJ. Accurate identification is crucial, especially in cases that are refractory to conventional therapy [3].

Magnetic resonance imaging (MRI) remains the preferred modality for evaluating sacroiliitis, offering high sensitivity for bone marrow edema and other inflammatory or degenerative changes [4–7]. However, clinical correlation is critical, as imaging findings alone may not fully explain symptom severity [8].

Treatment options include pharmacologic agents, physiotherapy, and image-guided intra-articular injections [9, 10]. While conservative measures are the first line of management, patients with persistent symptoms may benefit from targeted interventions. CT-guided SIJ injection provides precise needle placement and high technical success, especially in anatomically challenging cases [11, 12].

Despite the increasing use of CT guidance, comparative data on its effectiveness for inflammatory versus degenerative causes are limited. Furthermore, the impact of patient factors, such as age and body mass index (BMI), on treatment outcomes remains unclear.

Therefore, we aimed to assess the safety, technical success, and clinical outcomes of CT-guided SIJ injection in patients with chronic sacroiliitis and to examine how disease etiology, age, and BMI influence treatment response.

## Materials and methods

### Study design and ethical approval

This retrospective cohort study was conducted at a single tertiary care center between February 2020 and May 2022. The study was approved by the Institutional Review Board of Kafrelsheikh University (approval number: KFSIRB200-402). All patients provided written informed consent before the procedure.

### Patient selection

**Fig. 1:**
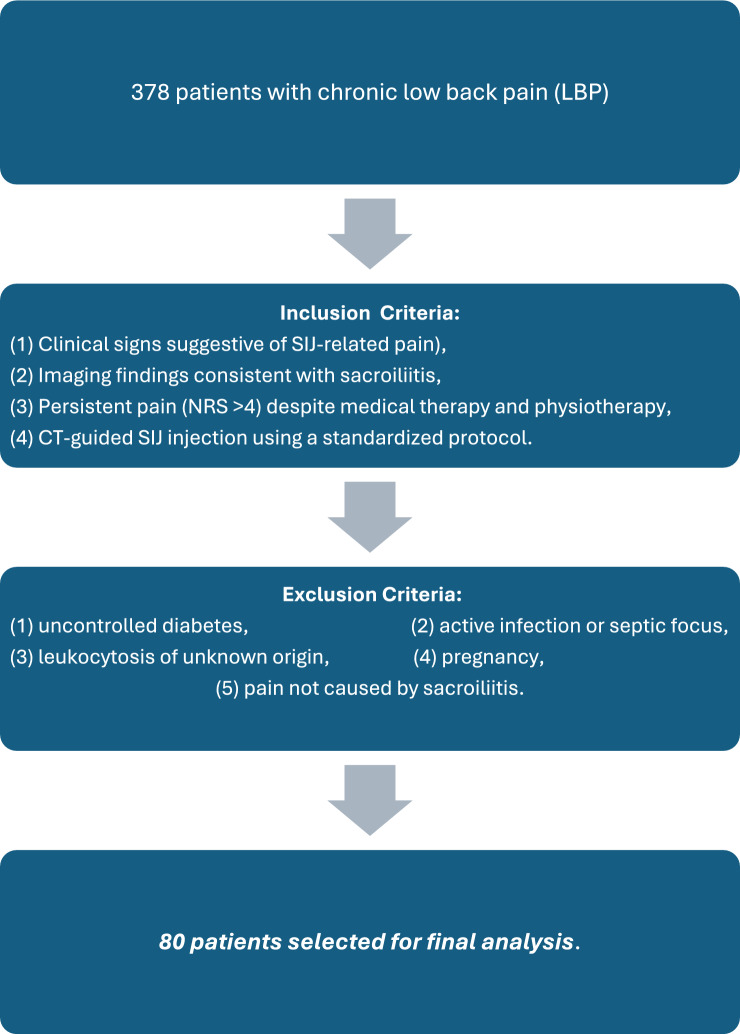
Flowchart of patient selection. Of the 378 patients with chronic low back pain screened for sacroiliac joint (SIJ) injection, 80 met the inclusion criteria and were enrolled

A total of 378 patients with chronic low back pain (LBP) were screened for sacroiliac joint (SIJ) injection during the study period. *Of these, 80 patients met the inclusion criteria and were retrospectively selected for analysis* (Fig. 1).

#### Inclusion and exclusion criteria

Patients were included if they: (1) had clinical signs suggestive of SIJ-related pain (e.g., positive provocation tests), (2) had imaging findings consistent with sacroiliitis, (3) reported persistent pain (NRS > 4) despite medical therapy and physiotherapy, and (4) underwent CT-guided SIJ injection using a standardized protocol. *These criteria, which reflect standard clinical protocols at our institution, were applied during retrospective chart review to ensure a consistent study population.*

On the other hand, exclusion criteria included patients with: (1) uncontrolled diabetes, (2) active infection or septic focus, (3) leukocytosis of unknown origin, (4) pregnancy, and (5) pain not caused by sacroiliitis.

### Clinical and imaging assessment

All patients underwent a detailed clinical evaluation, including a comprehensive history, physical examination, and provocation tests (Patrick, gapping, and sacral thrust). Imaging included pelvic X-ray and MRI in selected cases. Laboratory workup included HLA-B27, rheumatoid factor, ESR, CRP, CBC, and INR. *All imaging was reviewed by two radiologists who were blinded to the clinical data to minimize bias.*

### Procedure protocol

**Fig. 2:**
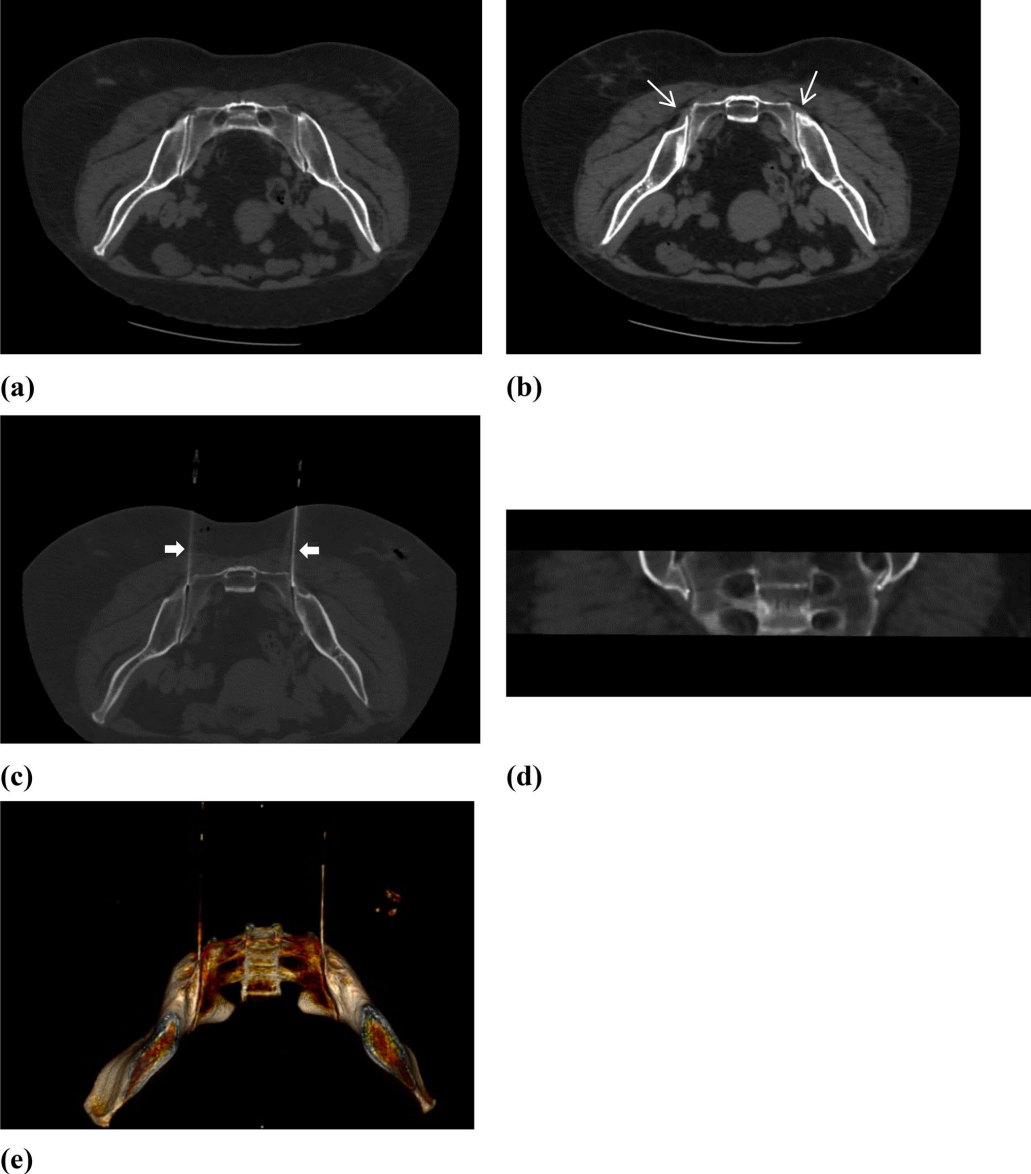
CT-guided SIJ injection in a 27-year-old female with bilateral inflammatory sacroiliitis. No degenerative changes are noted. Axial images show capsule distension (**a** and **b**), needle placement within the inferior joint compartments (**c**), and confirmation via a coronal image (**d**) and 3D reconstruction (**e**)

**Fig. 3:**
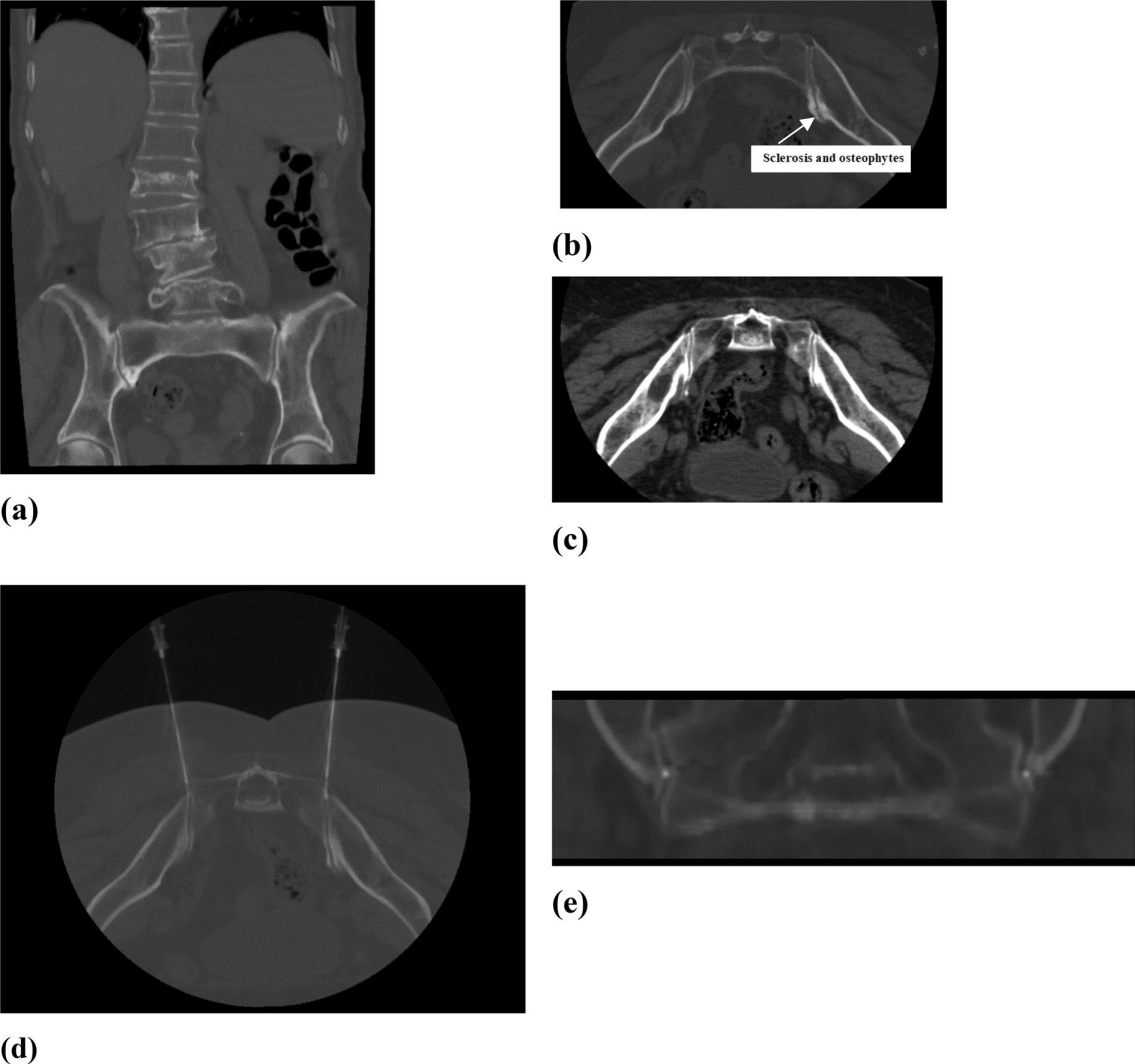
CT-guided SIJ injection in a 56-year-old female with lumbar scoliosis in the coronal plane (**a**), accompanied by secondary bilateral degenerative sacroiliitis, more common on the right side, with sclerosis and osteophytes, and no capsule distension seen in axial images (**b** and **c**). The procedure involved successful needle targeting (**d**). Image quality in (**e**) is lower due to the use of a low-dose acquisition technique to reduce radiation exposure

**Fig. 4:**
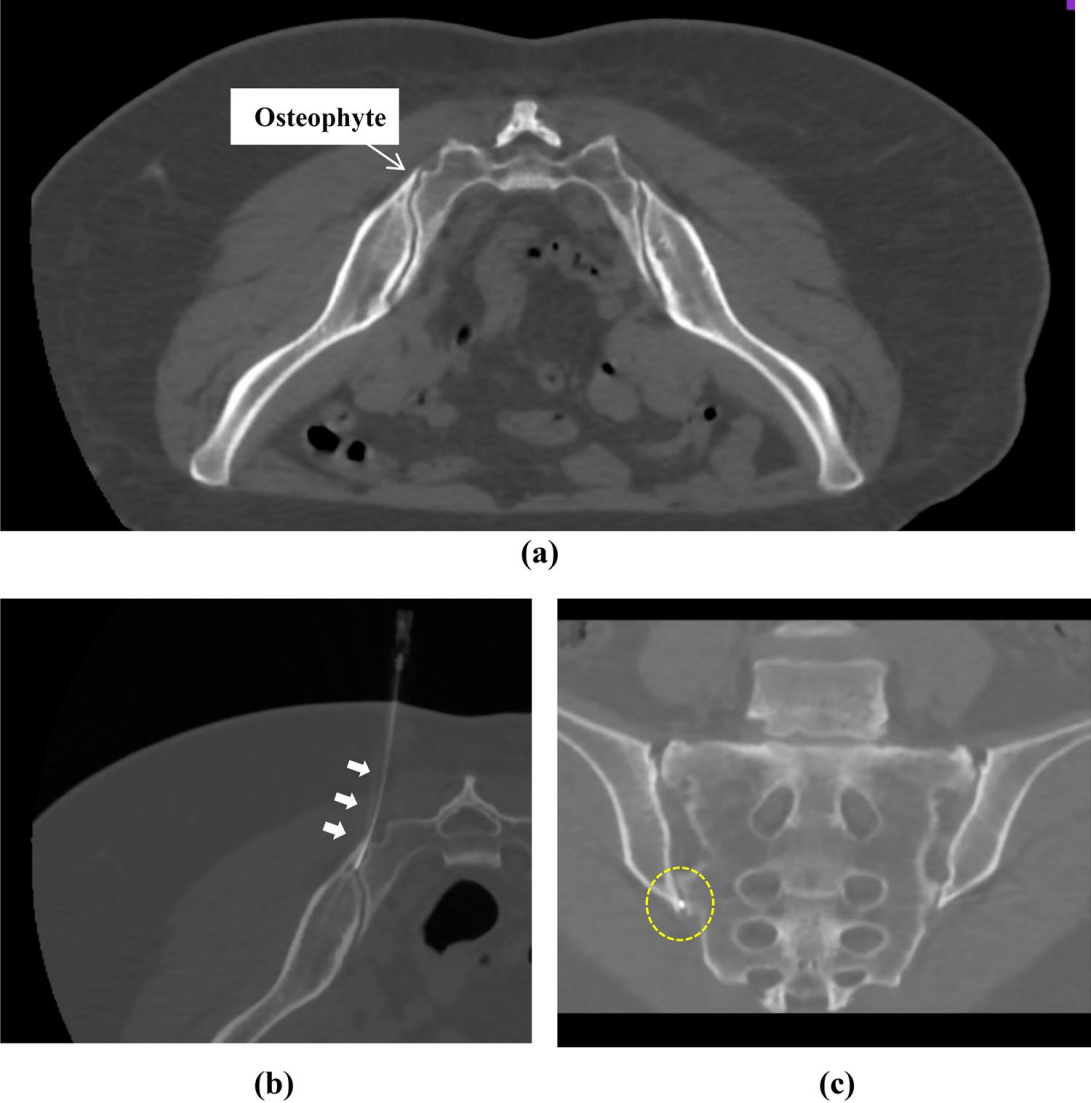
CT-guided SIJ injection performed on a 60-year-old female with left-sided symptomatic degenerative sacroiliitis. A prominent osteophyte (arrow in **a**) necessitated an oblique needle trajectory (short arrows in **b**), confirmed by coronal reconstruction correlation (dotted circle in **c**)

*All procedures were performed by a senior interventional radiologist with 20 years of experience, assisted by two radiologists with 7 years of experience each.* All CT-guided sacroiliac joint (SIJ) injections were done on an outpatient basis under a low-dose protocol (80 kV, 50 mAs). Following an initial localizing CT scan (with a 2 mm slice thickness) to plan the trajectory and mark the entry point, the skin was prepared and draped using aseptic technique. Subcutaneous local anesthesia was administered using 1–2 mL of 2% lidocaine. A 22-gauge spinal needle was then advanced under intermittent CT guidance to target the posterior inferior synovial compartment of the joint. The correct intra-articular needle tip position was confirmed on axial and, if necessary, coronal reconstructed images before proceeding (Figs. 2, 3 and 4). After negative aspiration to exclude intravascular placement, a therapeutic injectate mixture of 0.5 mL hydrocortisone, 0.5 mL lidocaine, and 1 mL bupivacaine was slowly administered. During needle withdrawal, an additional 3 mL of 2% lidocaine was injected peri-articularly to provide short-term analgesic coverage and minimize corticosteroid tracking. Patients were monitored for one hour post-procedure before discharge.

### Follow-up and outcome measures

Patients were assessed at 1 week, 1 month, and 6 months following the procedure using the Numerical Rating Scale (NRS, 0–10) and the Oswestry Disability Index (ODI, 0–100) to evaluate pain improvements in comparison with baseline values. *To reduce confounding variables, no additional analgesics or physiotherapy were administered during the follow-up period. Clinical success was defined as a reduction of 50% or more in NRS scores from baseline*, with the ODI serving as a supplementary measure of functional improvement.

### Statistical analysis

Data were analyzed using IBM SPSS software package version 20.0 (IBM Corp, Armonk, NY, USA). The Kolmogorov–Smirnov test was used to verify the normality of the variable distributions. The Friedman test was applied to compare more than two periods or stages of abnormally distributed quantitative variables, and Dunn's post hoc test was used for pairwise comparisons. The Mann–Whitney test was used to compare two groups with abnormally distributed quantitative variables. The Kruskal–Wallis test was used to compare more than two studied groups with abnormally distributed quantitative variables, and Dunn's post hoc test was used for pairwise comparisons. Significance of the obtained results was set at the 5% level.

## Results

### Patient characteristics

Eighty patients (52 females, 28 males; mean age 51.6 ± 9.7 years) were included. *All participants underwent CT-guided SIJ injections according to standardized clinical and imaging protocols.* Sacroiliitis was bilateral in 63.8% and unilateral in 36.3%. Pain was localized in 31.3% and referred in 68.8%, most commonly to the gluteal region and posterior thigh (Tables 1 and 2).

**Table 1:** Distribution of studied cases according to patient characteristics (*n* = 80)

	No. (%)
Age (years)	
Mean ± SD	51.64 ± 9.66
Median (min.–max.)	50 (32–72)
Sex	
Male	28 (35.0%)
Female	52 (65.0%)
Type	
Degenerative	53 (66.3%)
Inflammatory	27 (33.8%)
+ ve RF & HLA-B27	7 (25.9%)
− ve RF & HLA-B27	15 (55.6%)
Crohn’s disease	5 (18.5%)
BMI (kg/m^2^)	
Mean ± SD	26.48 ± 4.42
Median (min.–max.)	25 (20–38)

*SD* standard deviation

**Table 2:** Distribution of cases according to clinical findings (*n* = 80)

Side	
Unilateral	29 (36.3%)
Bilateral	51 (63.8%)
Symmetrical	26 (51.0%)
More on the right	15 (29.4%)
More on the left	10 (19.6%)
Pain	
Localized	25 (31.3%)
Referred	55 (68.8%)
Inferior gluteal & back upper thigh	29 (52.7%)
Anterior upper thigh	4 (7.3%)
Inguinal	5 (9.1%)
Combined	15 (27.3%)
Whole posterior thigh	2 (3.6%)
Trigger	
Unknown	7 (8.8%)
Stand up	29 (36.3%)
Sitting bending	26 (32.5%)
Walking	18 (22.5%)

Etiologically, 53 patients (66.3%) had degenerative sacroiliitis, and 27 (33.8%) had inflammatory disease. Among the inflammatory cases, 55.6% had elevated CRP/ESR with negative HLA-B27 and RF; 25.9% were positive for HLA-B27 and RF; and 18.5% had Crohn’s disease (Table 1).

### Procedural safety and technical success

*All procedures were technically successful, defined as intra-articular delivery of the full injectate volume confirmed by CT.* No complications were reported among our patients. The radiation dose ranged from 15 to 27 mGy.cm^2^ using a low-dose protocol.

### Clinical outcomes

All patients achieved clinical success, *as defined by a reduction of at least 50% in the Numeric Rating Scale (NRS), at the six-month follow-up*. The NRS scores decreased from 6.86 ± 1.20 at baseline to 2.26 ± 0.78 after six months (*p* < 0.001), while the Oswestry Disability Index (ODI) scores declined from 37.28 ± 9.84 to 5.68 ± 2.49 over the same period (*p* < 0.001) (Table 3). The ODI was employed as a supplementary metric for assessing functional improvement.

**Table 3:** Comparison between the studied periods according to NRS and ODI

	Before	1 Week	1 Month	6 Months	Fr	*p*
NRS						
Mean ± SD	6.86 ± 1.20	1.80 ± 0.85	1.84 ± 0.80	2.26 ± 0.78	177.39^*^	< 0.001^*^
Sig. bet. periods (*p*_1_)		< 0.001^*^	< 0.001^*^	< 0.001^*^		
ODI						
Mean ± SD	37.28 ± 9.84	6.18 ± 2.80	5.21 ± 2.39	5.68 ± 2.49	180.198^*^	< 0.001^*^
Sig. bet. Periods (*p*_1_)		< 0.001^*^	< 0.001^*^	< 0.001^*^		

*SD* standard deviation, *Fr* Friedman test, Sig. bet. periods using Dunn’s post hoc test, *p* value for comparing between the different studied periods, *p*_1_ value for comparing before treatment and each other period, *statistical significance at *p* ≤ 0.05

### Subgroup analysis by etiology

Both degenerative and inflammatory subgroups demonstrated significant improvement. The reduction in NRS was comparable between the groups at all time points (*p* > 0.05). However, the improvement in ODI was more pronounced in inflammatory cases at 1-month and 6-month follow-up (*p* < 0.05), indicating greater functional recovery (Table 4). *These differences, although statistically significant, were modest in magnitude and should be interpreted with caution.*

**Table 4:** Evolution of NRS and ODI by sacroiliitis types (*n* = 80)

		Type		*U*	*p*
		Degenerative (*n* = 53)	Inflammatory (*n* = 27)		
NRS	Before				
	Mean ± SD	6.98 ± 1.23	6.63 ± 1.11	612.50	0.280
	1 Week				
	Mean ± SD	1.81 ± 0.88	1.78 ± 0.80	694.00	0.814
	1 Month				
	Mean ± SD	1.89 ± 0.75	1.74 ± 0.90	648.0	0.445
	6 Months				
	Mean ± SD	2.30 ± 0.75	2.19 ± 0.83	658.5	0.530
ODI	Before				
	Mean ± SD	40.04 ± 9.28	31.85 ± 8.70	335.0^*^	< 0.001^*^
	1 Week				
	Mean ± SD	6.45 ± 2.87	5.63 ± 2.63	615.5	0.298
	1 Month				
	Mean ± SD	5.72 ± 2.51	4.22 ± 1.80	421.5^*^	0.002^*^
	6 Months				
	Mean ± SD	6.19 ± 2.63	4.67 ± 1.86	507.0^*^	0.030^*^

*SD* standard deviation, *U* Mann–Whitney test, *p* value for comparing between degenerative and inflammatory types, *statistical significance at *p* ≤ 0.05

### Subgroup analysis by BMI and age

Patients were stratified by BMI: less than 25 (*n* = 32), 25–30 (*n* = 34), and greater than 30 (*n* = 14). No significant differences in NRS or ODI were observed between BMI groups at any follow-up point (*p* > 0.05) (Table 5). An age-based analysis indicated that patients over 50 years of age exhibited higher baseline ODI scores. Although ODI remained elevated in older patients at follow-up, all age groups showed statistically significant improvements, and age did not significantly influence NRS response (Tables 6 and 7). *Due to the limited sample sizes within these subgroups, the findings should be regarded as preliminary and exploratory.*

**Table 5:** Evolution of NRS and ODI by BMI (*n* = 80)

		BMI (kg/m^2^)			*H*	*p*
		< 25 (*n* = 32)	25 – 30 (*n* = 34)	> 30 (*n* = 14)		
NRS	Before					
	Mean ± SD	6.78 ± 1.10	6.79 ± 1.32	7.21 ± 1.12	2.179	0.336
	1 Week					
	Mean ± SD	1.72 ± 0.92	1.71 ± 0.76	2.21 ± 0.80	3.511	0.173
	1 Month					
	Mean ± SD	1.91^a^ ± 0.93	1.59^ab^ ± 0.70	2.29^a^ ± 0.47	9.538^*^	0.008^*^
	6 Months					
	Mean ± SD	2.25 ± 0.88	2.18 ± 0.72	2.50 ± 0.65	2.042	0.360
ODI	Before					
	Mean ± SD	34.97 ± 9.90	38.50 ± 9.97	39.57 ± 8.88	3.923	0.141
	1 Week					
	Mean ± SD	5.59 ± 2.65	6.44 ± 2.79	6.86 ± 3.11	1.853	0.396
	1 Month					
	Mean ± SD	5.16 ± 2.62	5.26 ± 2.45	5.21 ± 1.76	0.352	0.839
	6 Months					
	Mean ± SD	5.72 ± 2.90	5.62 ± 2.34	5.71 ± 1.98	0.005	0.997

H, Kruskal–Wallis test, pairwise comparison between each 2 group using Dunn’s post hoc test for multiple comparisons

*SD* standard deviation; *p* value for comparing between the different categories; *statistical significance at *p* ≤ 0.05; means with common letters are not significantly different (i.e., means with different letters are significant)

**Table 6:** Evolution of NRS and ODI by age (*n* = 80)

		Age (years)		*U*	*p*
		≤ 50 (*n* = 42)	> 50 (*n* = 38)		
NRS	Before				
	Mean ± SD	6.79 ± 1.05	6.95 ± 1.35	766.0	0.751
	1 Week				
	Mean ± SD	1.81 ± 0.89	1.79 ± 0.81	784.5	0.889
	1 Month				
	Mean ± SD	1.79 ± 0.87	1.89 ± 0.73	726.0	0.441
	6 Months				
	Mean ± SD	2.12 ± 0.83	2.42 ± 0.68	638.5	0.096
ODI	Before				
	Mean ± SD	34.33 ± 9.07	40.53 ± 9.74	491.0^*^	0.003^*^
	1 Week				
	Mean ± SD	5.86 ± 2.82	6.53 ± 2.78	700.0	0.334
	1 Month				
	Mean ± SD	4.55 ± 1.86	5.95 ± 2.70	549.5^*^	0.014^*^
	6 Months				
	Mean ± SD	5.0 ± 2.21	6.42 ± 2.61	586.0^*^	0.037^*^

*SD*, standard deviation; *U*, Mann–Whitney test; *p* value for comparing the different categories

*statistical significance at *p* ≤ 0.05

**Table 7:** Evolution of NRS and ODI with age correlation factors (*n* = 80)

		Age (years)	
		*r*	*p*
NRS	Before	0.198	0.079
	1 Week	0.003	0.976
	1 Month	0.100	0.377
	6 Months	0.275^*^	0.014^*^
ODI	Before	0.481^*^	< 0.001^*^
	1 Week	0.219	0.051
	1 Month	0.430^*^	< 0.001^*^
	6 Months	0.461^*^	< 0.001^*^

*r* Pearson coefficient

^*^statistical significance at *p* ≤ 0.05

## Discussion

Sacroiliac joint (SIJ) dysfunction is increasingly recognized as a key factor in chronic low back pain, especially in patients with previous lumbar spine surgery or persistent symptoms [1, 2, 13]. In this retrospective cohort study, CT-guided SIJ injection demonstrated high technical success and resulted in lasting clinical improvement in both inflammatory and degenerative conditions, consistent with previous research [14–17].

### Clinical outcomes and interpretation

Pain and disability scores improved significantly over the six-month period. All patients achieved at least a 50% reduction in NRS and notable functional improvements on the ODI. *Whereas the NRS was used to define clinical success, the ODI provided a complementary measure of functional recovery.* This dual assessment captures both symptom relief and the effect on daily activities. *The magnitude of improvement in our cohort aligns with prior reports on image-guided SIJ injections* [14, 18, 19]*. However, our cohort demonstrated a lower baseline disability, possibly reflecting an earlier intervention in the disease course.*

A key finding was the differential functional improvement between etiologies. Inflammatory cases demonstrated a statistically significant reduction in ODI at both 1- and 6-month follow-ups, consistent with prior reports of pronounced steroid responsiveness in inflammatory sacroiliitis [14, 18–20]. *However, the absolute differences were modest, and the robust response observed in degenerative cases confirms that CT-guided injection is broadly effective regardless of the underlying etiology*.

### Technical success and context

Our 100% technical success rate without complications underscores the precision of CT guidance. *This high technical success is a recognized advantage of CT over other modalities, such as ultrasonography, where reported miss rates can be significant* [21]*.* We employed low-dose protocols to minimize radiation exposure, aligning with modern best practices [11, 22]. However, since *we did not perform a direct dosimetric comparison with other guidance methods, such as fluoroscopy or ultrasound, the relative radiation efficiency of our technique remains an area for future research.*

We acknowledge that our study has several limitations that should be considered when interpreting the results. First, *the retrospective design and the lack of a control group preclude definitive causal conclusions, and the potential influence of placebo effects or natural symptom fluctuations cannot be ruled out*. Second, *our subgroup analyses for BMI and age were likely underpowered due to small sample sizes; the lack of significant findings here should be considered exploratory*. *Therefore, future prospective studies with comparator arms (e.g., US- or fluoroscopy-guided injection) and longer follow-up are warranted to validate these findings, determine long-term efficacy, and refine patient selection criteria* [11, 18].

Nonetheless, our research reinforces the role of CT-guided sacroiliac joint injections by providing structured outcome data across various etiologies. *The study highlights consistent clinical benefits for both inflammatory and degenerative cases, achieved through a standardized technique and assessed with reproducible criteria.*

## Conclusion

CT-guided sacroiliac joint injection is a safe and effective intervention for chronic sacroiliitis, yielding sustained pain relief and functional improvement across diverse patient profiles. These findings support its use in selected patients who do not respond to conservative management.

## Abbreviations

SIJ: Sacroiliac joint
LBP: Low back pain
MRI: Magnetic resonance imaging
US: Ultrasonography
CT: Computed tomography
BMI: Body mass index
NRS: Numerical Rating Scale
HLA-B27: Human leukocyte antigen B27
ODI: Oswestry Disability Index
